# Low-Energy Mechanochemical Dry Coating for Improving the Flowability of Sodium Naproxen

**DOI:** 10.3390/ma19204405

**Published:** 2026-10-20

**Authors:** Mateusz Przywara, Karolina Maria Leś, Marcin Chutkowski

**Affiliations:** Department of Chemical and Process Engineering, Rzeszow University of Technology, al. Powstańców Warszawy 6, 35-029 Rzeszów, Polandmarcin.chutkowski@prz.edu.pl (M.C.)

**Keywords:** sodium naproxen, dry coating, powder flowability, silica nanoparticles, Aerosil^®^ R972, mechanochemical processing, V-type mixer, Jenike shear cell, cohesion, pharmaceutical excipients

## Abstract

**Highlights:**

- A patented dry coating method improved sodium naproxen powder flowability.
- Nanostructured silica (Aerosil^®^ R972) was used as an effective flow modifier.
- Modified powders showed reduced cohesion and improved flow indices.
- SEM analysis confirmed silica deposition on particle surfaces.
- The method is relatively simple and potentially relevant to pharmaceutical processing.

**Abstract:**

Sodium naproxen is a widely used nonsteroidal anti-inflammatory drug (NSAID) known for its therapeutic efficacy but also for its poor flowability, which complicates manufacturing processes. This study aimed to improve the flow properties of sodium naproxen through a patented mechanochemical dry coating method using nanostructured silica (Aerosil^®^ R972) as a flow modifier. The process was conducted in a modified V-type tumbler mixer under selected conditions, including a 7:1 ball-to-powder mass ratio, a 10-min mixing time, and a 10 rpm rotational speed. Flowability was evaluated using Carr’s indices, while mechanical properties were assessed via shear testing with a Jenike cell. Surface morphology was characterized by SEM. Results showed that the modified sodium naproxen samples demonstrated a substantial improvement in flow indices, a shift to an “easy-flowing” classification at higher consolidation stresses, and a marked reduction in cohesion compared to the unmodified material. SEM confirmed partial silica deposition on particle surfaces. These findings indicate that the applied method effectively improves the handling characteristics of sodium naproxen powders. The process is relatively simple, requires low energy, is potentially applicable at a larger scale, and shows potential for pharmaceutical powder processing requiring flow enhancement of cohesive active ingredients.

## 1. Introduction

The pharmaceutical industry, as well as the nutraceutical and dietary supplement sectors, is highly dependent on the processing of powdered materials. In fact, most solid dosage forms, including tablets, capsules, and granules, are produced from powders that must exhibit sufficient flowability to ensure efficient production and dose uniformity. Poor powder flow can result in significant manufacturing challenges, such as segregation, incomplete die filling, bridging, ratholing, and inconsistent drug content in final products [1,2,3,4].

Fine powders, especially those with particle sizes below 100 μm, such as sodium naproxen, a widely used nonsteroidal anti-inflammatory drug (NSAID), pose particular challenges in pharmaceutical processing due to their cohesive behavior, including spontaneous agglomeration, clumping, and wall adhesion. These phenomena affect powder flow, reduce process efficiency, and may compromise product quality [5,6,7]. Moreover, the flowability of powders is not only influenced by intrinsic properties such as particle size, shape and surface chemistry, but also by external factors, including humidity, temperature, and the material’s processing history, commonly referred to as the memory effect [2,8,9]. As a result, predictive theoretical models for powder flow under industrial conditions remain insufficient, and experimental evaluation using standardized methods is required [10].

Standard approaches to powder flow evaluation include the Carr index system, which assesses flowability based on parameters such as bulk and tapped density, compressibility, and angles of repose and fall [8,10]. More advanced techniques, such as direct shear testing employing the Jenike cell, yield the flow function coefficient (*ffc*), which characterizes powder flow behavior under higher stress and storage conditions [8,11]. The application of these methods provides quantitative insights into material behavior under realistic processing constraints.

In order to enhance flow properties, various strategies have been proposed, including granulation, process-based modifications, and, most notably, dry coating or the addition of flow-enhancing excipients, especially nanostructured additives such as silica, titanium dioxide, or magnesium stearate [12,13,14]. These additives adsorb onto the particles’ surfaces, thereby reducing van der Waals forces and internal friction and enhancing powder mobility [12,13,15]. However, challenges such as an inhomogeneous distribution of the additives and insufficient energy input for agglomerate disruption may still hinder the resulting flow performance [16,17].

Recent studies have demonstrated that intensive mechanical dry coating can effectively overcome these limitations [9,16]. This approach involves the use of high-energy mixing equipment such as hybridizers, mechanofusion devices, conical mills, or planetary ball mills, capable of generating sufficient shear and impact forces to achieve uniform dispersion of both the primary particles and the additives throughout the powder bed [17,18,19]. This technique offers a promising approach to flow improvement by promoting efficient coating and minimizing agglomerate formation [20].

Direct shear testing using the Jenike apparatus provides one of the most informative approaches for evaluating the flowability of cohesive pharmaceutical powders. By preconsolidating the powder bed and measuring the shear stress required to induce failure, the method yields parameters such as the yield locus, unconfined yield strength, and flow function coefficient, which together describe powder behavior under stress conditions relevant to storage, discharge, and feeding operations [11,21,22,23]. In contrast to simpler empirical indices, Jenike-based measurements are particularly valuable for materials prone to caking, bridging, arching, and wall adhesion because they capture flow behavior under low-stress conditions that more closely resemble industrial handling environments [5,7,11,21,24]. Accordingly, the Jenike apparatus is widely used not only to classify powders according to their flowability but also to support hopper design and process optimization for pharmaceutical materials with complex or highly cohesive flow behavior [5,11,21,23].

In the present study, the V-type tumbler mixer was mechanically intensified by introducing zirconia balls into the mixing chamber. The objective was to increase particle–particle and particle–media interactions while maintaining the relatively simple and low-speed operation of a conventional tumbler system. The novelty of the present approach therefore lies in the specific mechanically intensified mixer configuration and its application to the dry coating of highly cohesive sodium naproxen, rather than in the dry coating concept itself.

The equipment commonly employed for dry coating is mainly used at the laboratory scale, which limits the direct transfer of the process to industrial-scale applications. To enhance process scalability, the use of a conventional V-blender was proposed. However, the low mixing intensity was found to be insufficient for effective deagglomeration of the additive [16,25]. Therefore, in the present study, the V-blender was modified by introducing process-intensifying balls into the mixing chamber, transforming the low-intensity mixing process into a mechanically intensified dry coating process. The movement of the balls enhances the frequency and intensity of particle–particle interactions, promoting the deagglomeration of additive particles and their deposition onto the carrier particle surfaces. This may provide an important advantage in terms of simplifying equipment design and facilitating the scale-up of the dry coating process, as it does not require a high-speed rotor, fluidizing gas, or milling screen.

Nevertheless, optimizing the concentration of flow enhancers and mixing parameters remains crucial, as both overdosing and underdosing can negatively affect flow or induce segregation, electrostatic charging, or compromised dissolution [9,12,15]. Therefore, customized solutions tailored to the specific material and processing conditions are essential.

## 2. Materials and Methods

### 2.1. Materials

Highly cohesive sodium naproxen powder (Divi’s Laboratories Ltd., Hyderabad, India) was selected to improve its flow properties via the dry coating method. The particle size of sodium naproxen (host particles) is approximately 10-900 µm, and the moisture content is below 0.5%.

**Figure 1:**
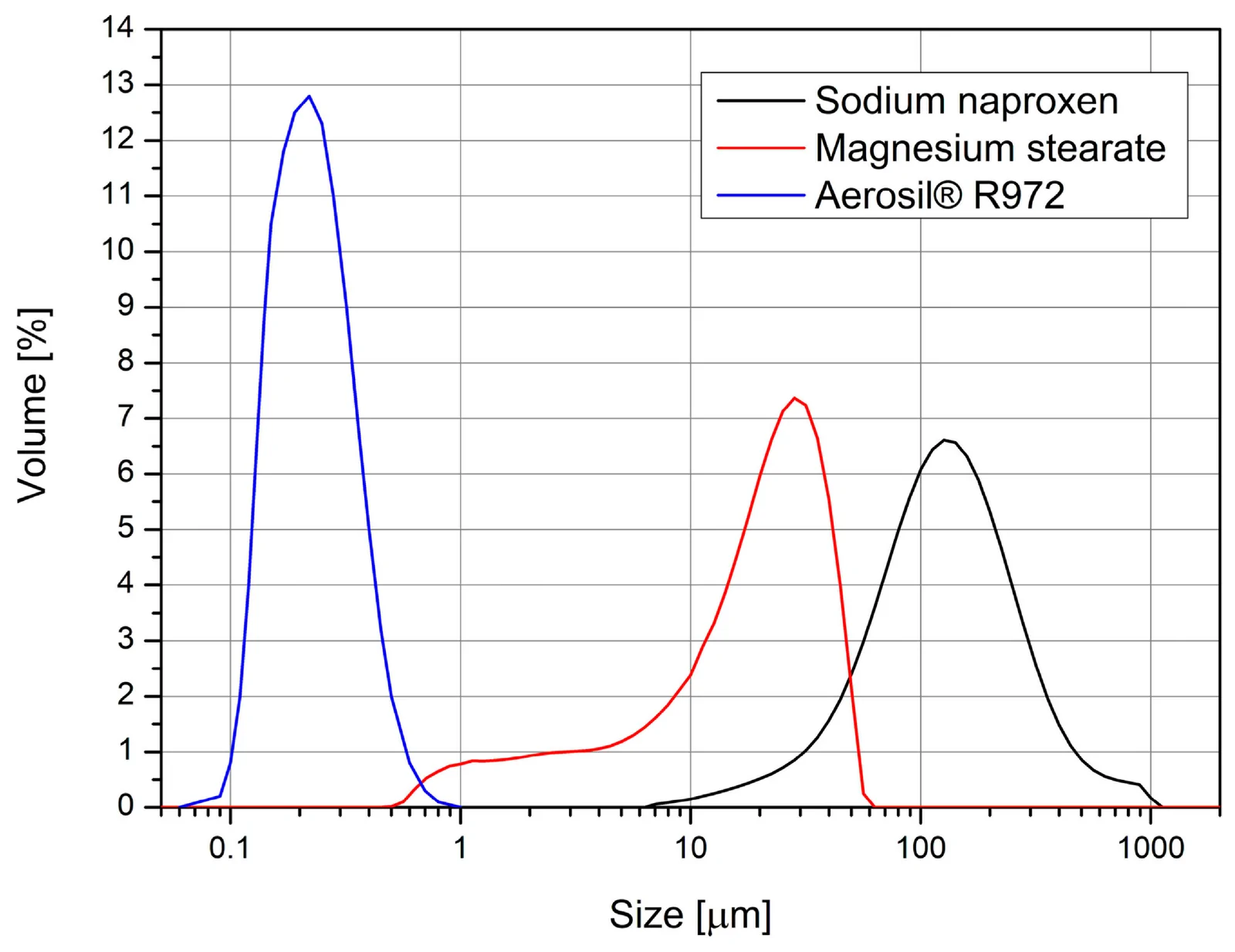
Particle size distribution of materials.

Based on preliminary experiments, common pharmaceutical excipients such as magnesium stearate from Sigma-Aldrich (St. Louis, MO, USA) and hydrophobic colloidal fumed silica Aerosil^®^ R972 (Evonik, Essen, Germany) were selected as modifiers. The particle size distributions of the proposed additives (guest particles), presented in Figure 1, fulfilled the requirements for the dry coating process, while the moisture content of both excipients was below 0.5%. Aerosil^®^ R972 exhibited a very fine and narrow particle size distribution, with the majority of particles located in the submicron range and a distinct peak at approximately 0.2–0.3 μm. In contrast, magnesium stearate showed a broader particle size distribution, with most particles in the range of several micrometers to several tens of micrometers and a maximum around 30 μm. The substantial difference in particle size between the modifiers and the host sodium naproxen particles is advantageous for dry coating, as the finer guest particles can effectively adhere to and cover the surface of larger host particles, potentially reducing interparticle cohesion and improving powder flowability. Various concentrations of the modifiers were investigated, taking into account the maximum levels permitted by the European Pharmacopoeia [26].

### 2.2. Interactive Mixing

The proposed approach to improving the flowability of sodium naproxen is dry coating via interactive mixing. The successful application of this technique requires the accurate selection of the coating methodology, appropriate additives, and the optimization of all process parameters.

**Figure 2:**
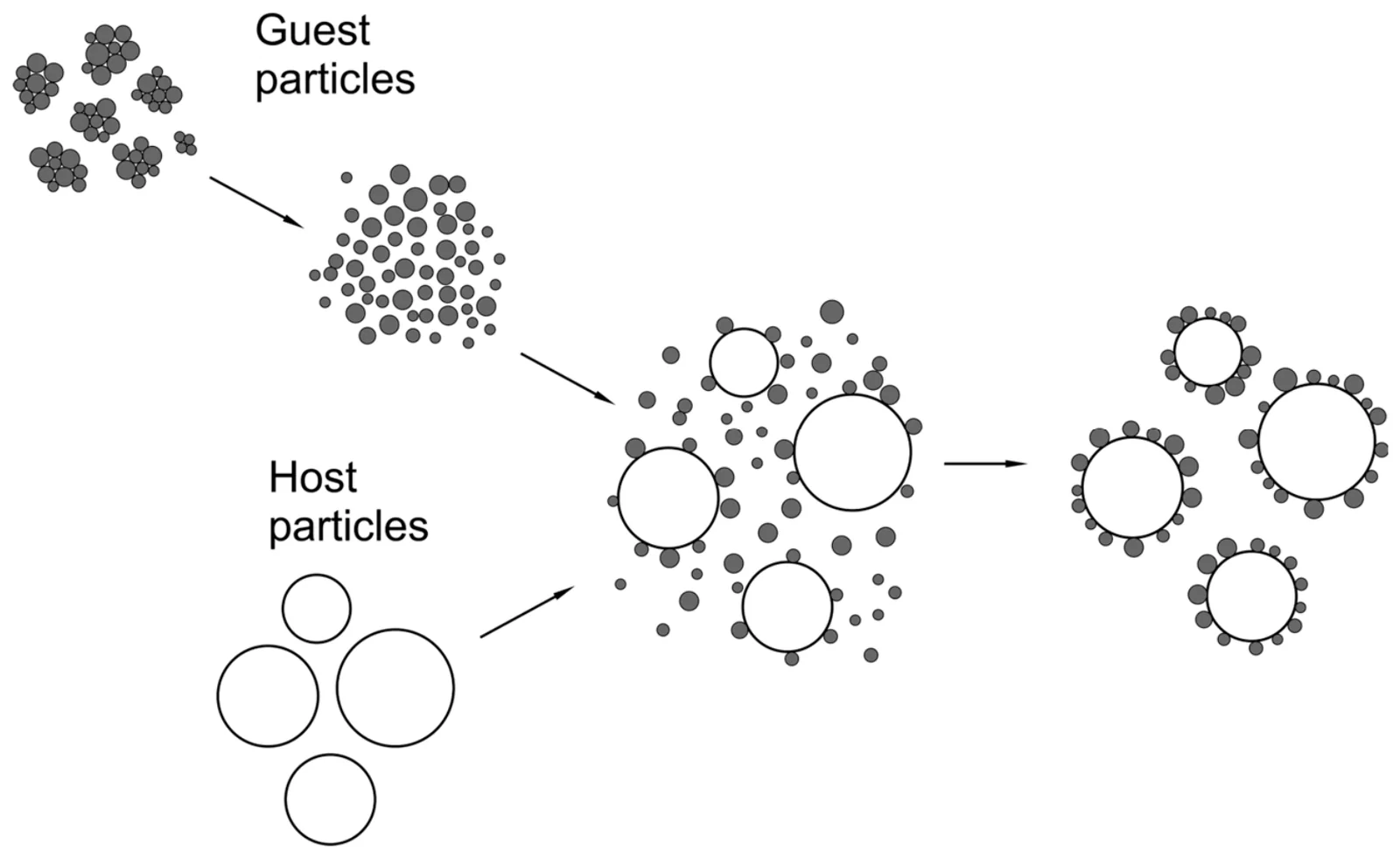
Schematic of dry coating.

The dry coating of powder particles relies on intermolecular forces and involves the formation of a thin layer of coating particles (guest particles) on the surface of the carrier particles (host particles)—Figure 2. The surface modification achieved through the addition of glidants reduces interactions between host particles, which enhances flow properties and eliminates many common issues associated with powder processing. This work proposed to improve powder flowability by depositing guest particles onto the surface of sodium naproxen particles while maintaining other processing parameters and the investigated powder properties.

**Figure 3:**
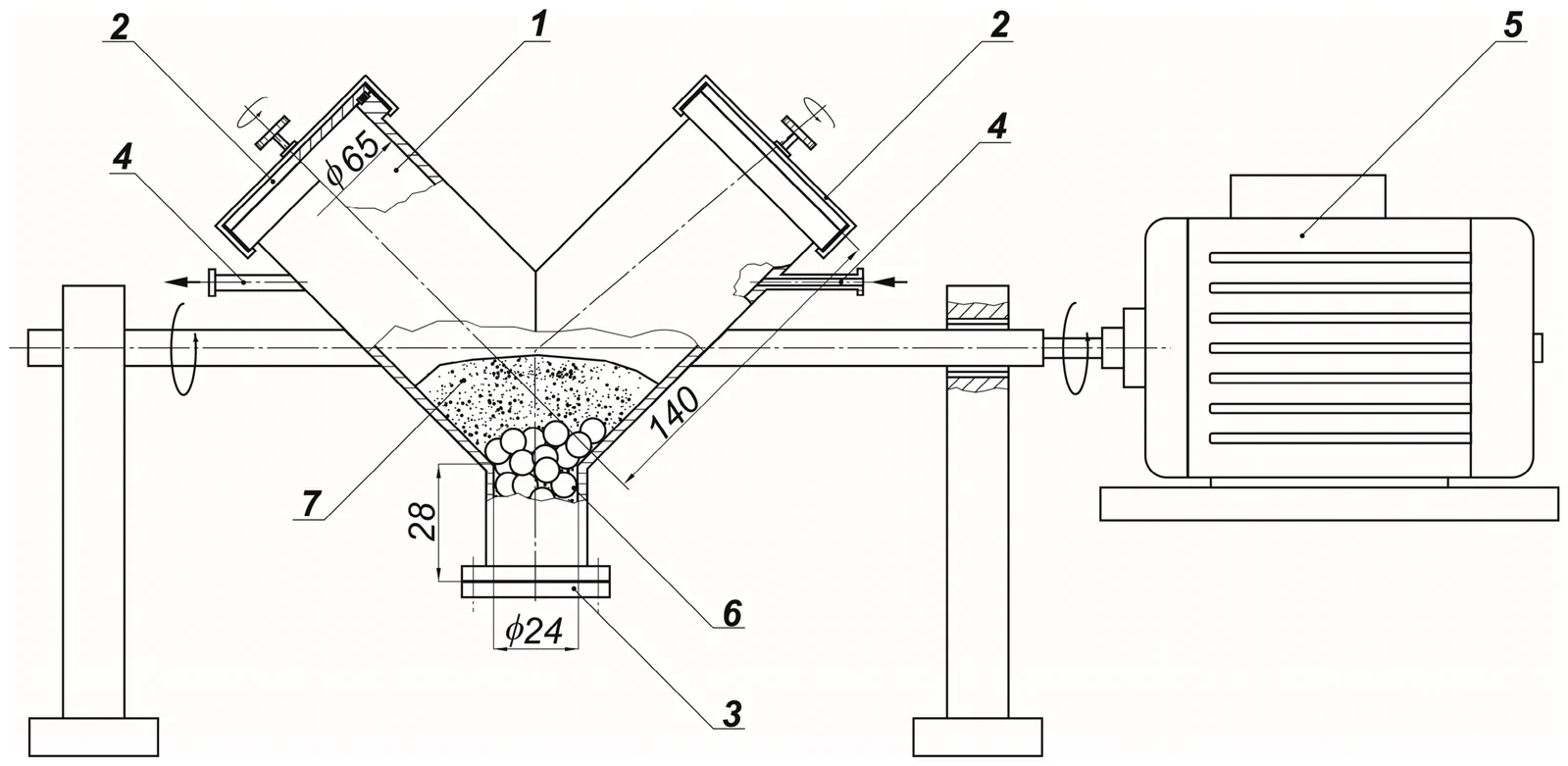
Diagram of the modified V-type tumbler mixer: 1—chamber; 2 and 3—cover; 4—air inlet and outlet; 5—drive system; 6—balls; 7—modified powder.

To improve the handling properties of sodium naproxen via dry coating, a custom-modified V-type tumbler mixer from CDK, Gliwice, Poland (800 cm^3^) was utilized (Figure 3). The mixer was equipped with zirconium oxide milling balls (5 mm diameter), applied at mass ratios of 4:1 and 7:1 relative to the powder mixture, as established in preliminary studies. Mixing was conducted at room temperature for 10 min at a rotational speed of 10 rpm, with the chamber filled to approximately 40% of its total volume (including both powder and milling media). The mixing time and rotational speed were selected based on preliminary screening experiments. These experiments indicated insufficient powder modification at shorter mixing times and lower rotational speeds, whereas prolonged mixing and higher rotational speeds increased the tendency toward powder adhesion or agglomeration. The ball-to-powder ratio was also preliminarily compared at 4:1 and 7:1, with the latter providing more favorable flowability and therefore being selected for subsequent experiments. In a typical procedure, pre-weighed zirconia balls, sodium naproxen, and the modifier were introduced into the sealed mixing chamber under the specified conditions. These parameters enabled efficient dry coating while reducing agglomeration and segregation. After processing, the powder was separated from the balls by sieving through a 1 mm mesh.

### 2.3. Sample Preparation and Flowability Assessment

The flowability of sodium naproxen and selected mixtures prepared according to Table 1 was investigated.

The flowability of the powders was evaluated based on selected Carr’s indices, such as angles of repose, fall, and difference, as well as compressibility index values, which were determined using a Powder Tester PT-S device (Hosokawa Micron B.V., Doetinchem, The Netherlands), following the ASTM D6393-14 standard [10] for dry powder samples. All measurements were performed in triplicate. The results are presented as mean ± standard deviation (SD), and the error bars shown in the figures represent SD. The angle of repose is defined as the maximum angle between the horizontal plane and the slope of a pile of granular material at which the material remains stable without sliding, while the angle of fall refers to the corresponding angle measured after three shocks delivered by an automatic shocker, causing partial rearrangement and settling of the particles. The angle of difference is defined as the numerical difference between the angle of repose and the angle of fall. The compressibility index *CI* is calculated based on the aerated (*ρ_a_*) and tapped (*ρ_t_*) bulk densities according to Equation (1):


(1) $$\mathrm{CI}=\frac{\rho_{t}-\rho_{a}}{\rho_{t}}\cdot 100\%$$

Aerated bulk density refers to the bulk density of a powder that has been loosely filled into a container, allowing a significant amount of air between particles. In contrast, packed bulk density is determined after mechanically tapping the container, usually with 180 taps, to compact the powder. During tapping, the powder becomes more densely packed, and this increased density is then measured.

A decrease in the angle of repose and compressibility index indicates an improvement in the flowability of the powder material. Conversely, a reduction in the angle of difference indicates a diminished propensity for avalanching.

Particle size distributions of sodium naproxen and magnesium stearate were determined by laser diffraction using a Mastersizer 2000 Hydro MU system (Malvern Instruments, Malvern, Worcestershire, UK) in accordance with ISO 13320-1:1999 [27]. Due to its significantly smaller particle size, the particle size distribution of Aerosil^®^ R972 was measured by dynamic light scattering (DLS) using a Zetasizer system (Malvern Instruments, Malvern, Worcestershire, UK).

The assessment of the mechanical properties of the powders began with the determination of the internal friction angle *φ*, the effective internal friction angle *φ_eff_*, and cohesion *C*. These measurements were conducted using the direct shear method with a Jenike shear tester (CDK, Gliwice, Poland), in accordance with the ASTM D6128 Standard Test Method for Shear Testing of Bulk Solids Using the Jenike Shear Tester [28].

The Jenike shear tester consists of two cylindrical shear cells, one of which is stationary while the other can move horizontally. According to the testing protocol, the apparatus is filled with a sample of the tested powder, subjected to a predetermined vertical consolidation load, and then sheared quasi-statically. During this pre-shear (consolidation) phase, the movable part of the cell is displaced at a very low velocity (approximately 1 mm/min). When the shear strength of the material is exceeded, the sample shears at the interface between the two parts of the cell and enters a state of plastic flow without further increase in shear stress.

Subsequently, the sample is reloaded with a reduced vertical normal stress, compared to the value applied during pre-shear, and subjected to another shearing step (actual shear) to induce plastic flow. This sequence of pre-shear and shear steps is repeated for progressively lower vertical normal stresses.

The results obtained from the shear tests are used to construct the material’s yield locus *YL*, from which key flow parameters are derived. This locus is typically represented by a straight line, referred to as the yield limit, yield line, flow limit line, or plastic flow line. The yield locus illustrates the relationship between the shear stress *τ_S_* at the onset of plastic flow and the applied normal stress *σ_N_*.

The slope of the yield line *YL* determines the internal friction angle *φ* of the material, while the cohesion *C* is defined by the intercept of this line with the shear stress axis *τ_S_*. The effective internal friction angle *φ*_eff_ is determined from the slope of the Effective Yield Locus *EYL*, i.e., a tangent line to the largest Mohr circle generated from the test data [5].

The flow function *FF* represents a functional relationship between the uniaxial compressive strength *σ_C_* and the major consolidation stress *σ*_1_, described by Equation (2):

*σ_C_* = *f*(*σ*_1_) (2)

This function allows for the classification of powder materials based on their resistance to gravity-driven free flow. Materials are categorized as free-flowing, easy-flowing, cohesive, or strongly cohesive. The flow function is widely used as an indicator of powder quality. It characterizes a powder’s tendency to hinder free gravitational flow under stress conditions that simulate bulk storage, such as in silos.

The classification of flowability is based on the flow factor index *ffc*, defined by Equation (3):

(3) $$\mathrm{ffc}=\frac{\sigma_{1}}{\sigma_{c}}$$

This index enables the assessment of the flow behavior of particulate materials using the following criteria:

- Free-flowing—*ffc* > 10
- Easy-flowing—10 > *ffc* > 4
- Cohesive—4 > *ffc* > 2
- Very cohesive, poorly flowing—2 > *ffc* > 1
- Extremely cohesive, very poorly flowing—*ffc* < 1

To determine the *FF*, it is necessary to construct multiple *YL* for powder samples subjected to various levels of consolidation stress.

The flow function *FF* and flowability index *ffc* of sodium naproxen samples were determined using a direct shear method with a Jenike shear cell. The procedure involved a series of pre-shear consolidation steps followed by shear testing under progressively lower normal stresses. For each measurement series, the powder bed was first consolidated under a defined normal load, after which shear tests were performed at several reduced normal stresses to determine the stress required to initiate material flow. This procedure was repeated for multiple levels of pre-shear consolidation in order to obtain a comprehensive description of the flow behavior under different stress conditions. The detailed measurement scheme is summarized in Table 2.

For each pre-shear consolidation load, shear measurements were performed at three different reduced normal loads, resulting in three experimental points used to construct each yield locus. These three points represent different normal stress levels and do not constitute independent experimental replicates. The complete Jenike measurement procedure was repeated three times for each tested material under the same experimental conditions. The results from the repeated measurement series were averaged, and the corresponding standard deviations were calculated.

Based on the experimental data, the corresponding *YL* and effective yield loci (*EYL*) were constructed. These were then used to determine the values of major consolidation stress *σ_1_* and uniaxial compressive strength *σ_C_*. The *FF* was presented as a graph depicting the relationship between *σ_C_* and *σ*_1_.

To evaluate changes on the surface of sodium naproxen after interactive mixing, high-resolution scanning electron microscopy (SEM) was employed. The analyses were conducted using an AURIGA CrossBeam Workstation scanning electron microscope (Carl Zeiss Microscopy GmbH, Oberkochen, Germany). The instrument operates within a primary electron beam energy range of 0.1–30 keV and provides a resolution of up to 1 nm at 30 keV. Its advanced detection capabilities enable surface imaging at electron energies as low as 100 eV. Prior to analysis, all powder samples were coated with a thin layer of gold.

## 3. Results

Based on the results of preliminary experiments, a recommended mixing time of 10 min was proposed. Shorter mixing durations led to insufficient dispersion of excipients on the surface of sodium naproxen, resulting in excipient agglomeration. Conversely, extended mixing times caused undesired adhesion of powder mixture particles to the walls of the mixer and the surface of the milling balls. A recommended rotational speed of 10 rpm was identified. Lower speeds failed to generate adequate mixing dynamics, whereas higher speeds resulted in material agglomeration.

**Figure 4:**
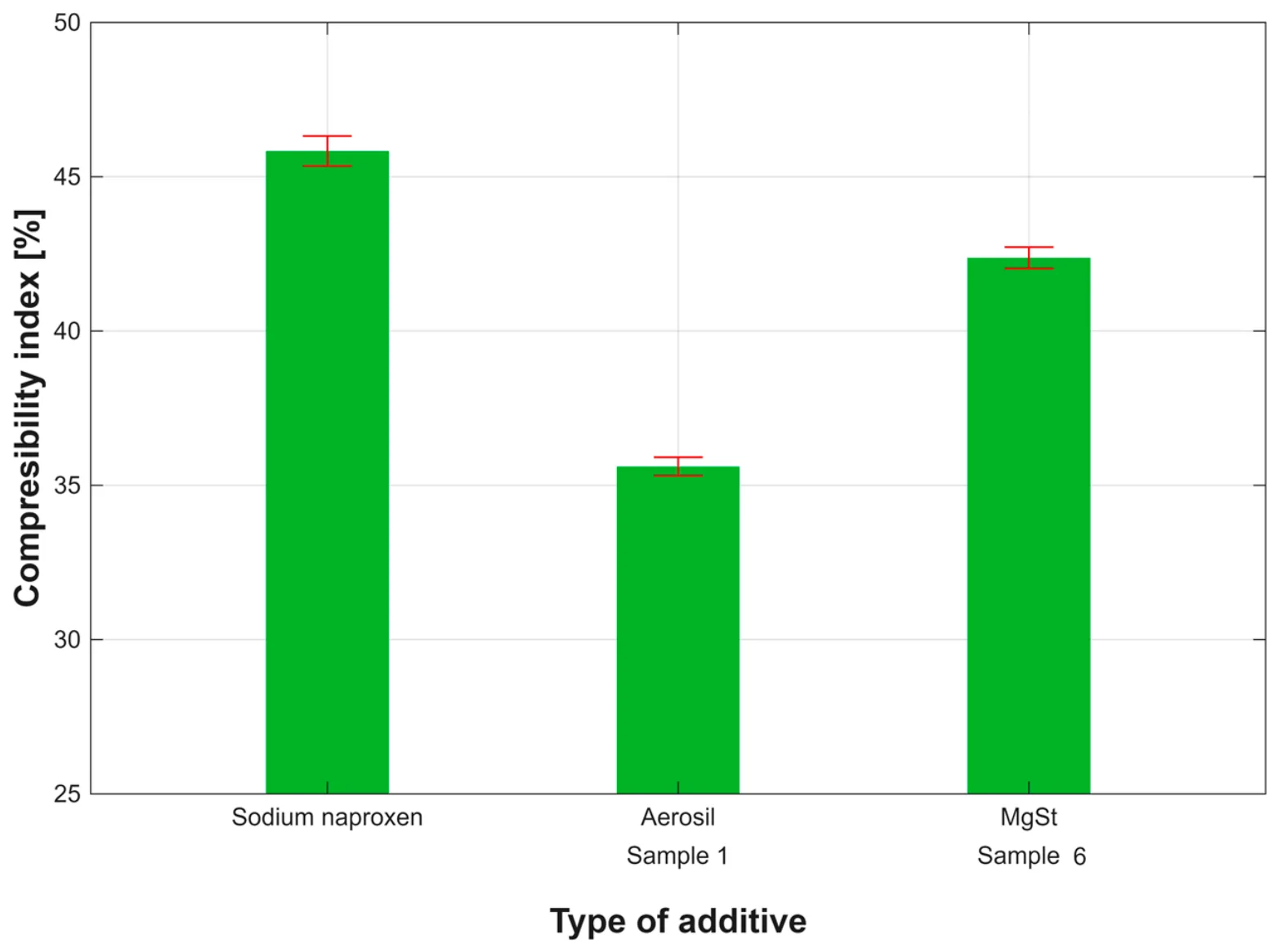
Compressibility index of unprocessed sodium naproxen, Sample 1 with Aerosil, and Sample 6 with magnesium stearate.

**Figure 5:**
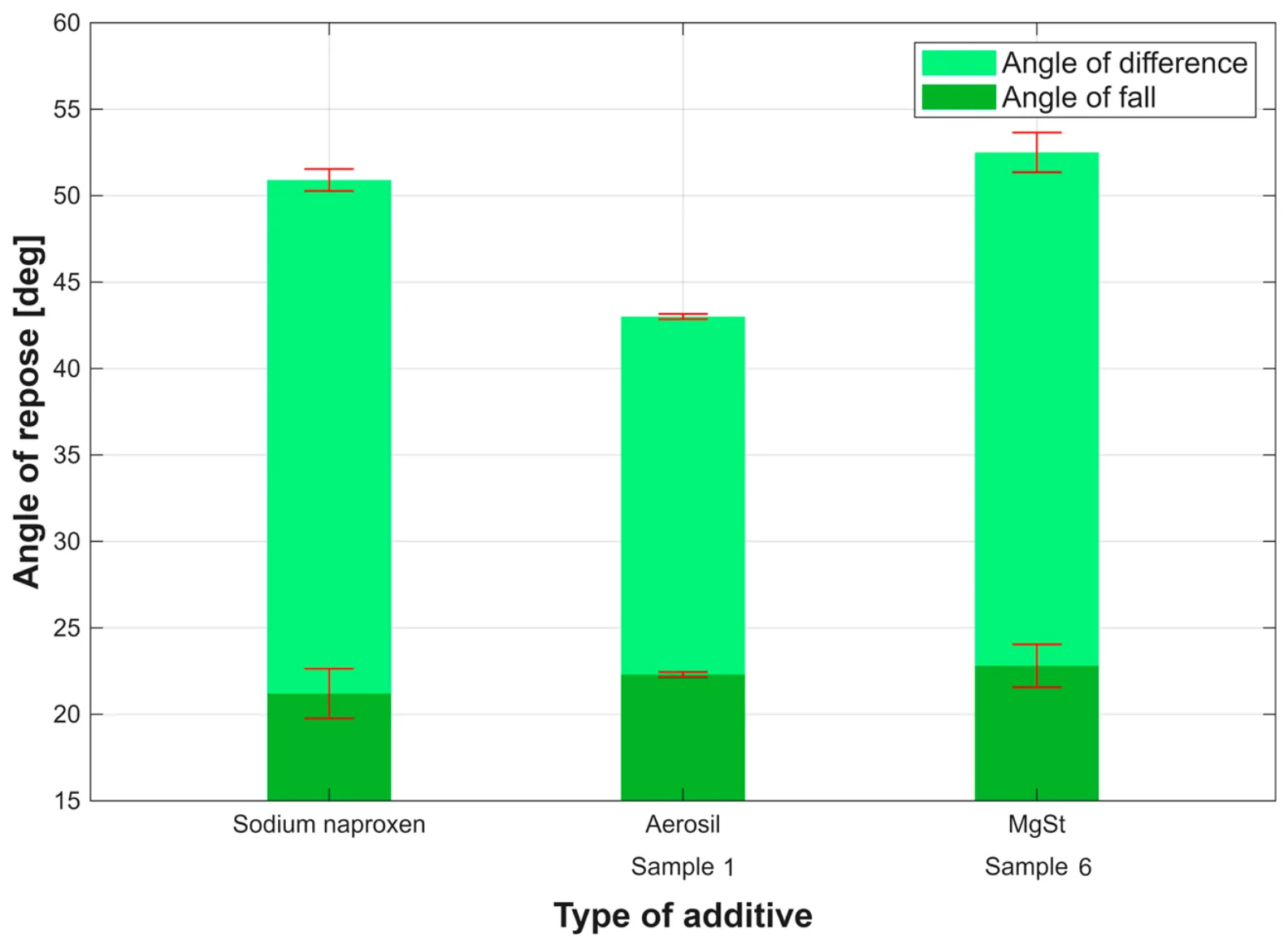
Angle of repose, fall and difference of unprocessed sodium naproxen, Sample 1 with Aerosil, and Sample 6 with magnesium stearate.

Most of the obtained samples showed an improvement in processability compared to unprocessed sodium naproxen (e.g., Sample 1 with Aerosil). A notable exception was Sample 6, which contained magnesium stearate as an excipient. Possibly due to the excessive particle size of this additive, no beneficial effect on the flowability of sodium naproxen was observed. The positive change in the mechanical properties of this sample was limited to a reduction in the compressibility index (Figure 4), which resulted from better packing of particles in the mixture with the finer-grained additive. However, the angle of repose of the modified sample was even greater than that of the unmodified sodium naproxen (Figure 5).

**Figure 6:**
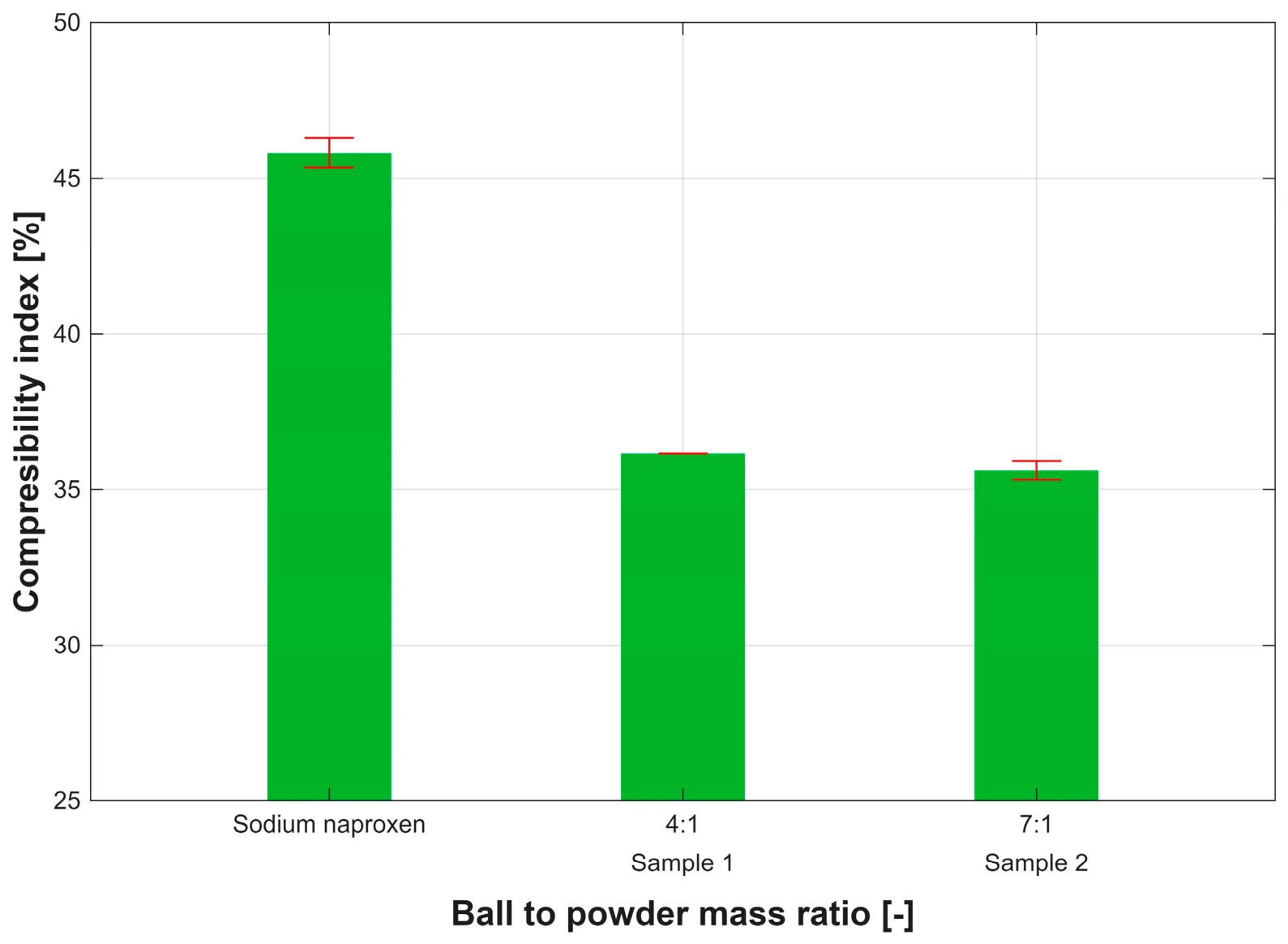
Compressibility index of unprocessed sodium naproxen, Sample 1, and Sample 2.

**Figure 7:**
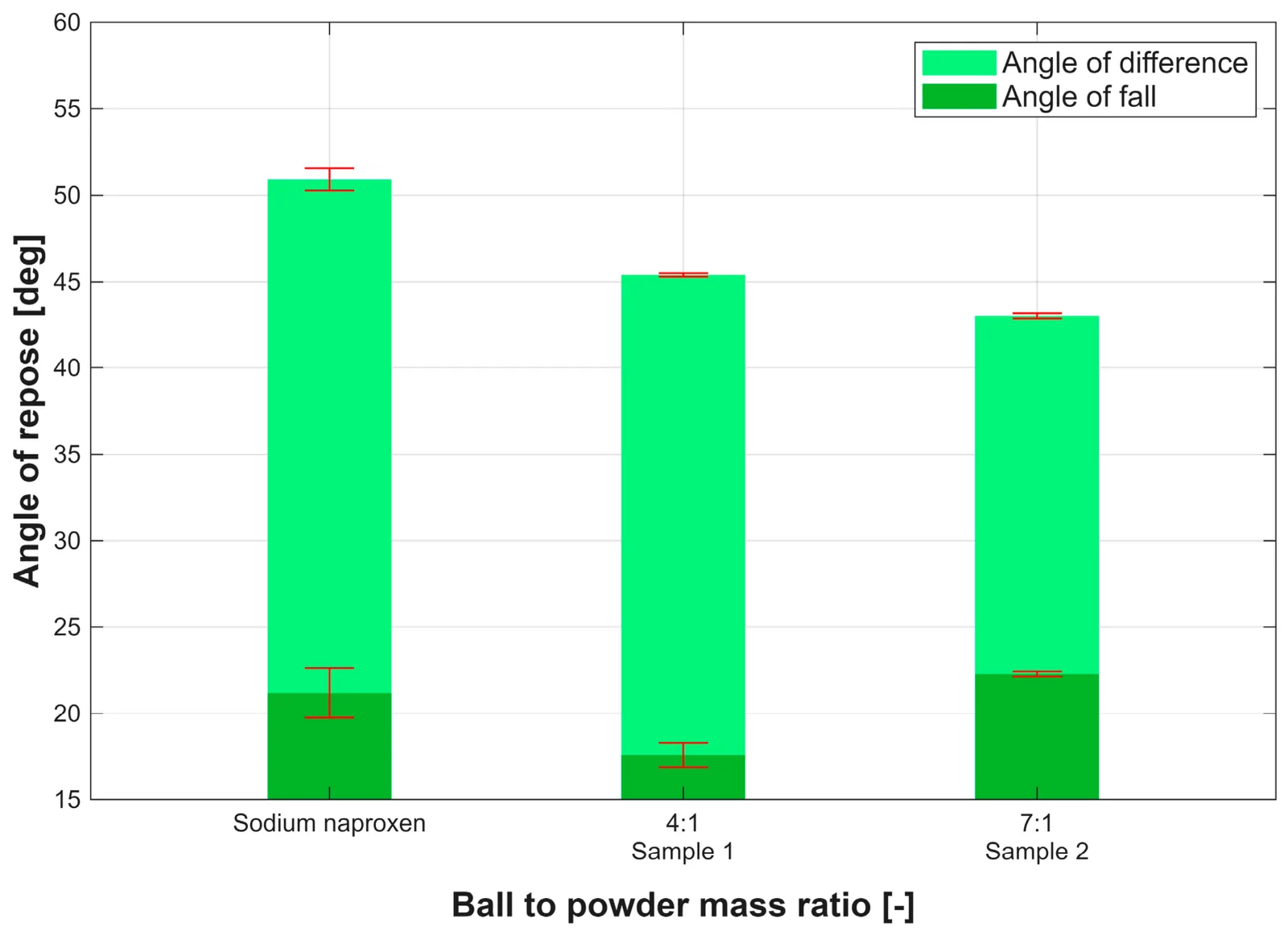
Angle of repose, fall and difference of unprocessed sodium naproxen, Sample 1, and Sample 2.

The use of a higher ball-to-powder mass ratio resulted in lower values of the compressibility index (Figure 6), as well as a lower angle of repose and angle of difference (Figure 7). These results indicate that Sample 1 exhibits better flowability and a reduced tendency toward avalanche discharge compared to Sample 2. Therefore, subsequent experiments were conducted using a ball-to-powder mass ratio of 7:1. A higher ball-to-powder ratio increases the intensity of mechanical interactions and energy transfer, which can promote powder deagglomeration and improve coating coverage.

**Figure 8:**
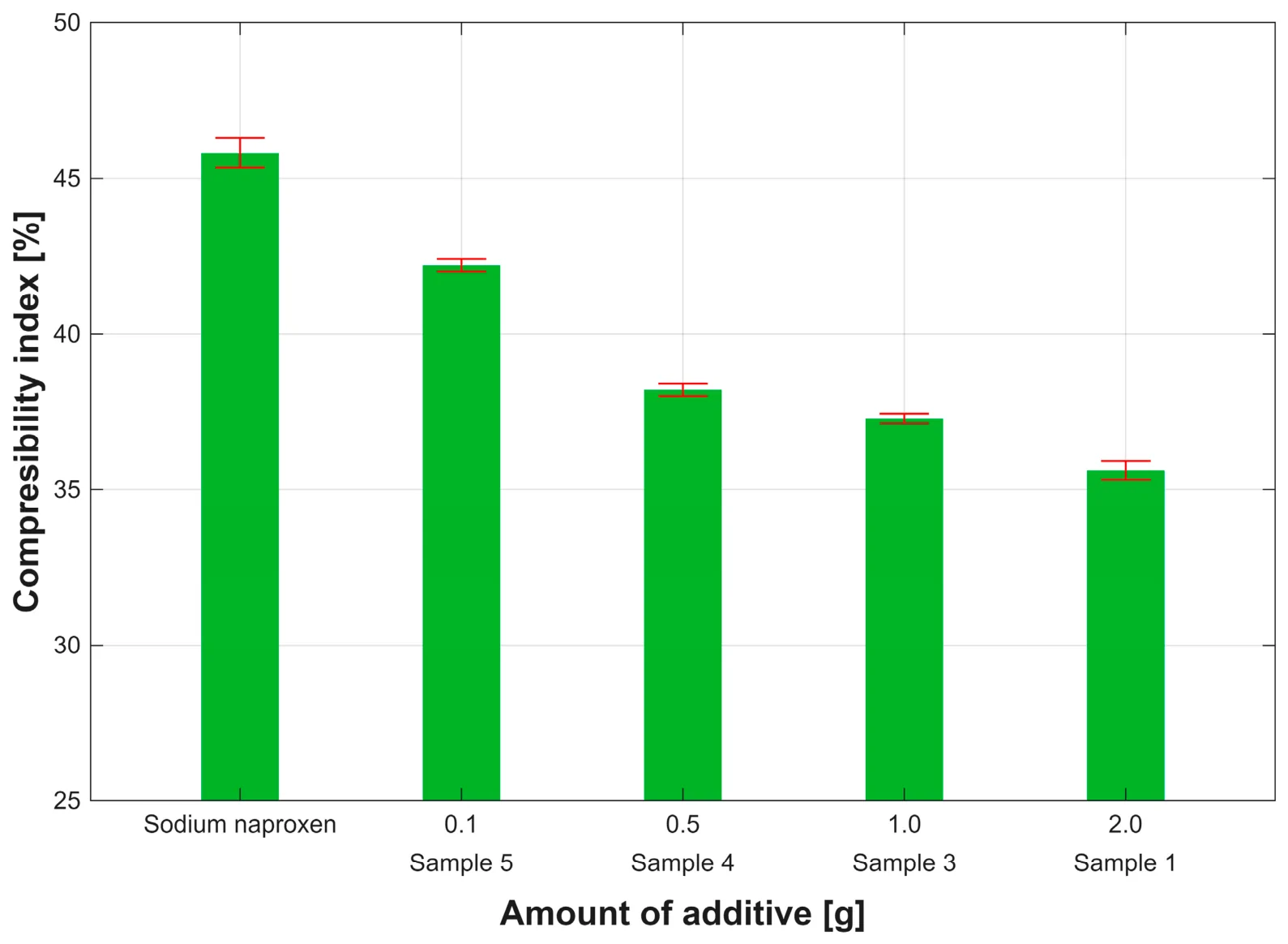
Compressibility index of unprocessed sodium naproxen and Samples 1 and 3–5.

**Figure 9:**
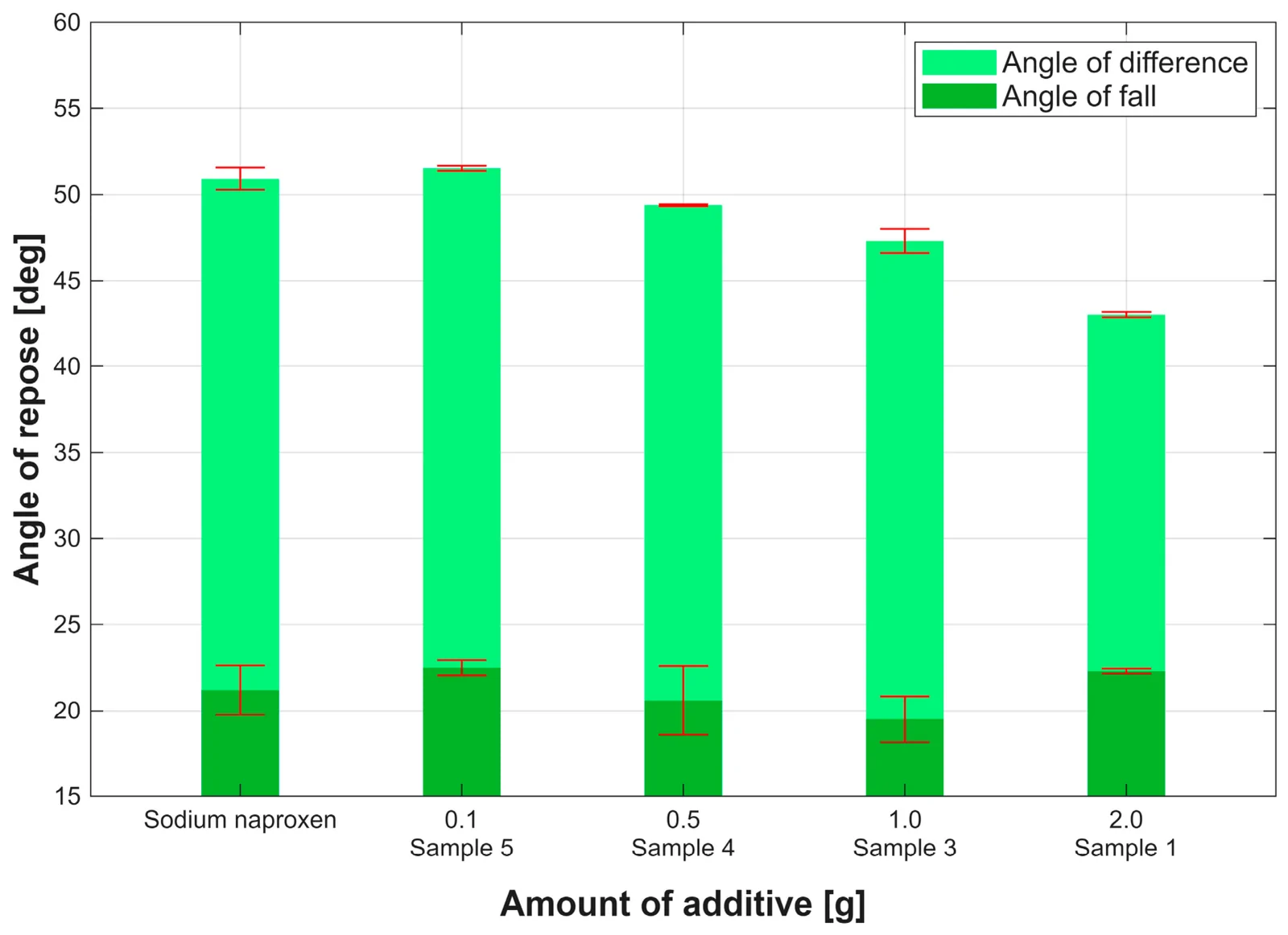
Angle of repose, fall and difference of unprocessed sodium naproxen and Samples 1 and 3–5.

Based on the flowability parameters measured for mixtures of sodium naproxen and Aerosil^®^ R972 produced using the modified V-type tumbler mixer, a marked decrease in the compressibility index was observed with increasing amounts of the modifier (Figure 8). This improvement in the packing properties of the powder was accompanied by a reduction in both the angle of repose and the angle of difference (Figure 9). Collectively, these results indicate enhanced flowability and, consequently, improved processability, which is highly desirable for industrial applications.

Unmodified sodium naproxen and the sample exhibiting the most favorable flow properties, i.e., Sample 1, characterized by a decrease in Carr indices indicative of improved powder flowability, were prepared in appropriate quantities (at least 2000 cm^3^ per sample) and subjected to shear tests using a Jenike shear cell.

The outcome of these tests was the determination of the material’s flow function, from which the following mechanical properties were derived: internal friction angle, effective internal friction angle, cohesion, uniaxial compressive strength, uniaxial and isostatic tensile strength, and maximum consolidation stress, as well as the flow index and flow function (Table 3 and Table 4).

**Figure 10:**
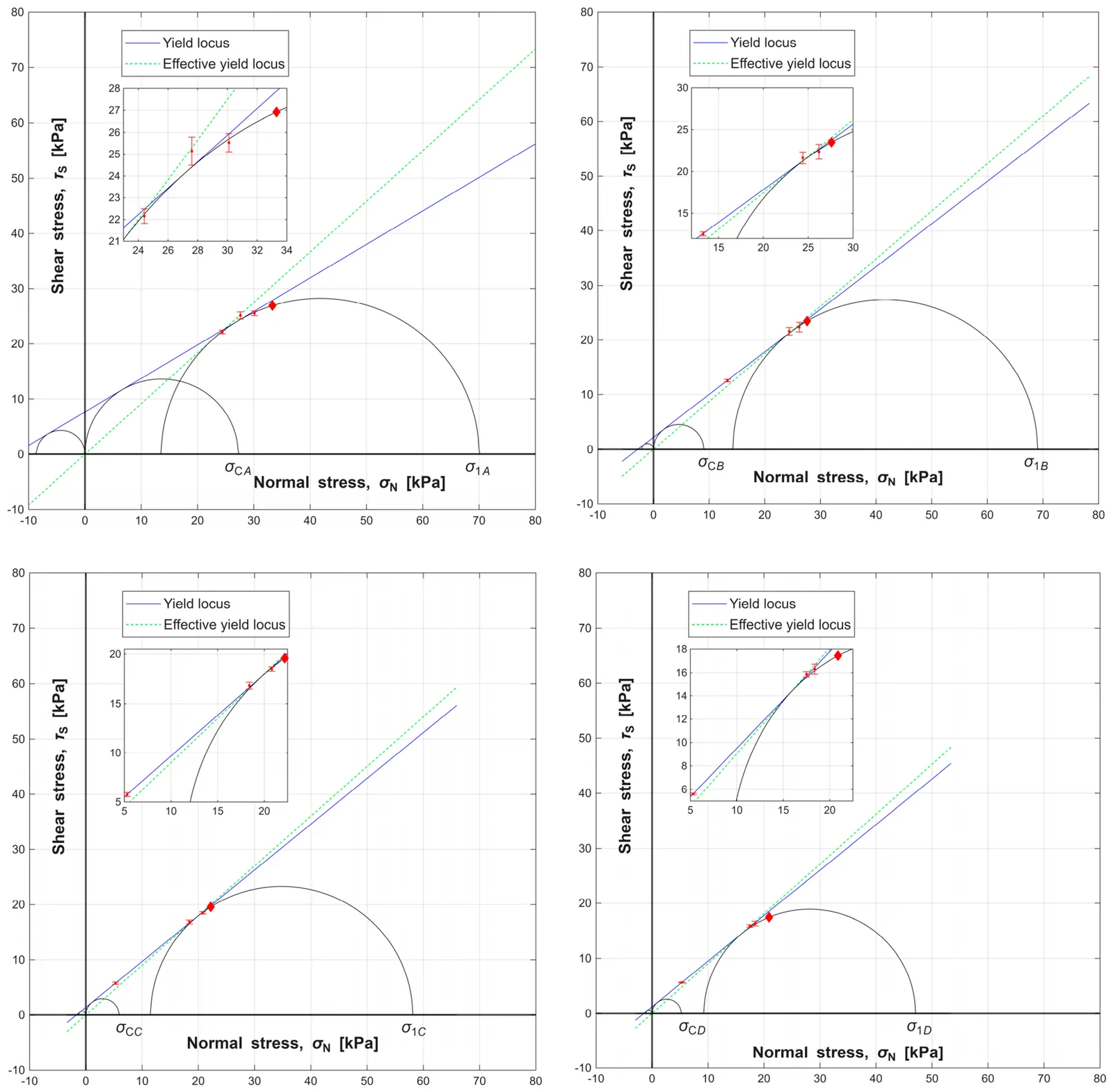
Yield loci (YL) and effective yield loci (EYL) of unmodified sodium naproxen determined at different pre-shear consolidation stresses. Red markers indicate the experimental shear points used for determination of the yield loci.

**Figure 11:**
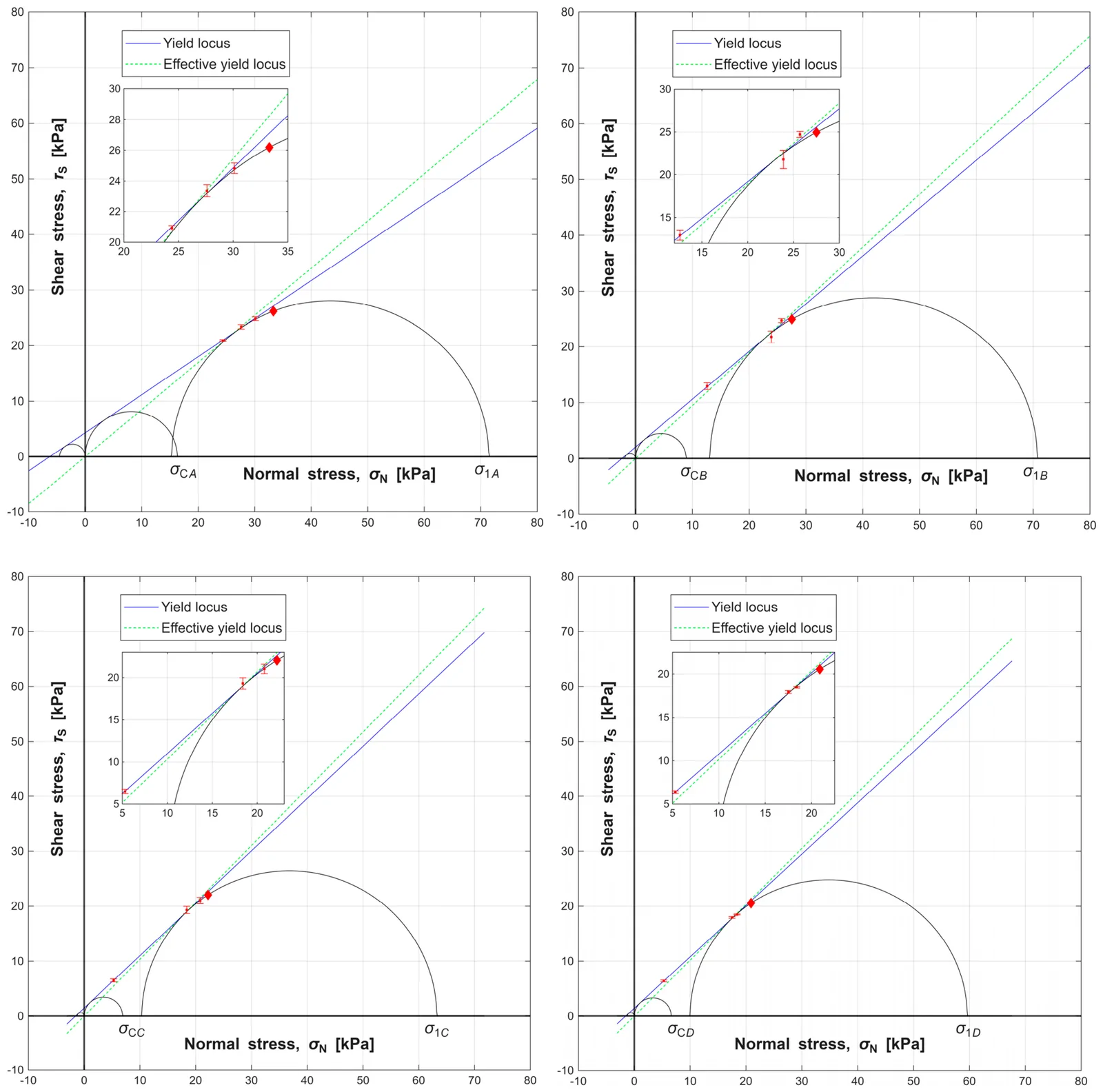
Yield loci (YL) and effective yield loci (EYL) of sodium naproxen modified with 2% Aerosil^®^ R972 (Sample 1) determined at different pre-shear consolidation stresses. Red markers indicate the experimental shear points used for determination of the yield loci.

Based on the conducted tests for sodium naproxen and Sample 1, selected samples exhibiting improved flowability, as confirmed by Carr indices, it was determined that under the applied normal consolidation stresses (20.9–33.3 kPa), both the modified material obtained via dry coating (Sample 1) and the unmodified sodium naproxen demonstrated similar strength characteristics (Figure 10 and Figure 11). The internal friction angle *φ* of the tested materials was found to decrease with increasing consolidation stress (*σ*_pre_).

However, this trend occurs within a lower range for unmodified sodium naproxen, with values decreasing from 39.2° to 31.26°, while the modified material (Sample 1) followed a comparable trend but within a slightly higher range, approximately 41–43° down to around 34–36°.

Regarding cohesion, an increase in this parameter was observed with rising consolidation stress (*σ*_pre_) for the modified sample. Below a consolidation load of 30 kPa, cohesion gently increased from approximately 1.2–1.4 kPa to around 1.5–2.2 kPa. A more pronounced difference between the raw and modified sodium naproxen became evident at the highest applied consolidation stress (*σ*_preD_ = 33.3 kPa): the modified material exhibited cohesion values between 3.3 and 4.3 kPa, whereas the unmodified material displayed nearly double that, with cohesion reaching 7.66 kPa.

**Figure 12:**
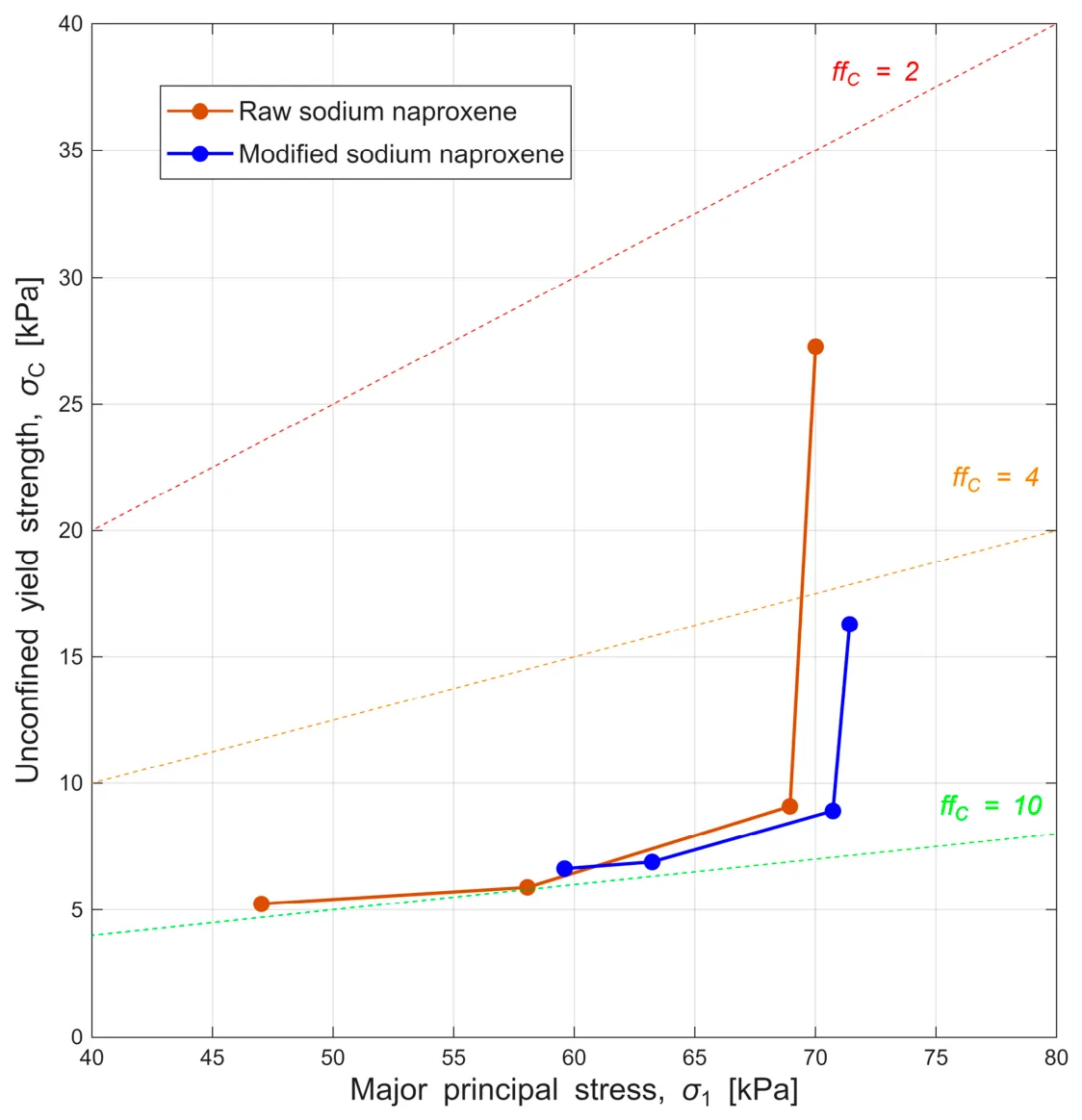
Flow functions of unmodified and mechanochemically modified sodium naproxen with 2% Aerosil^®^ R972 (Sample 1). The dashed lines indicate the flowability classification boundaries corresponding to *ffc* = 2, 4, and 10.

A comparison of the flow function (*FF*) for the samples showed similar overall behavior (Figure 12). The main distinction lies in a substantial increase in uniaxial compressive strength at the highest consolidation stress, which was observed for the unmodified sodium naproxen compared to the modified sample.

Therefore, it can be concluded that the applied mechanochemical modification did not substantially affect the strength characteristics of the samples under stresses below *σ*_1_ = 65 kPa. However, it did result in an increased flow index (*ffc*) at stresses above *σ*_1_ = 65 kPa, with values clearly exceeding *ffc* = 4. This indicates an improvement in the flowability of the modified material under higher stress conditions.

**Figure 13:**
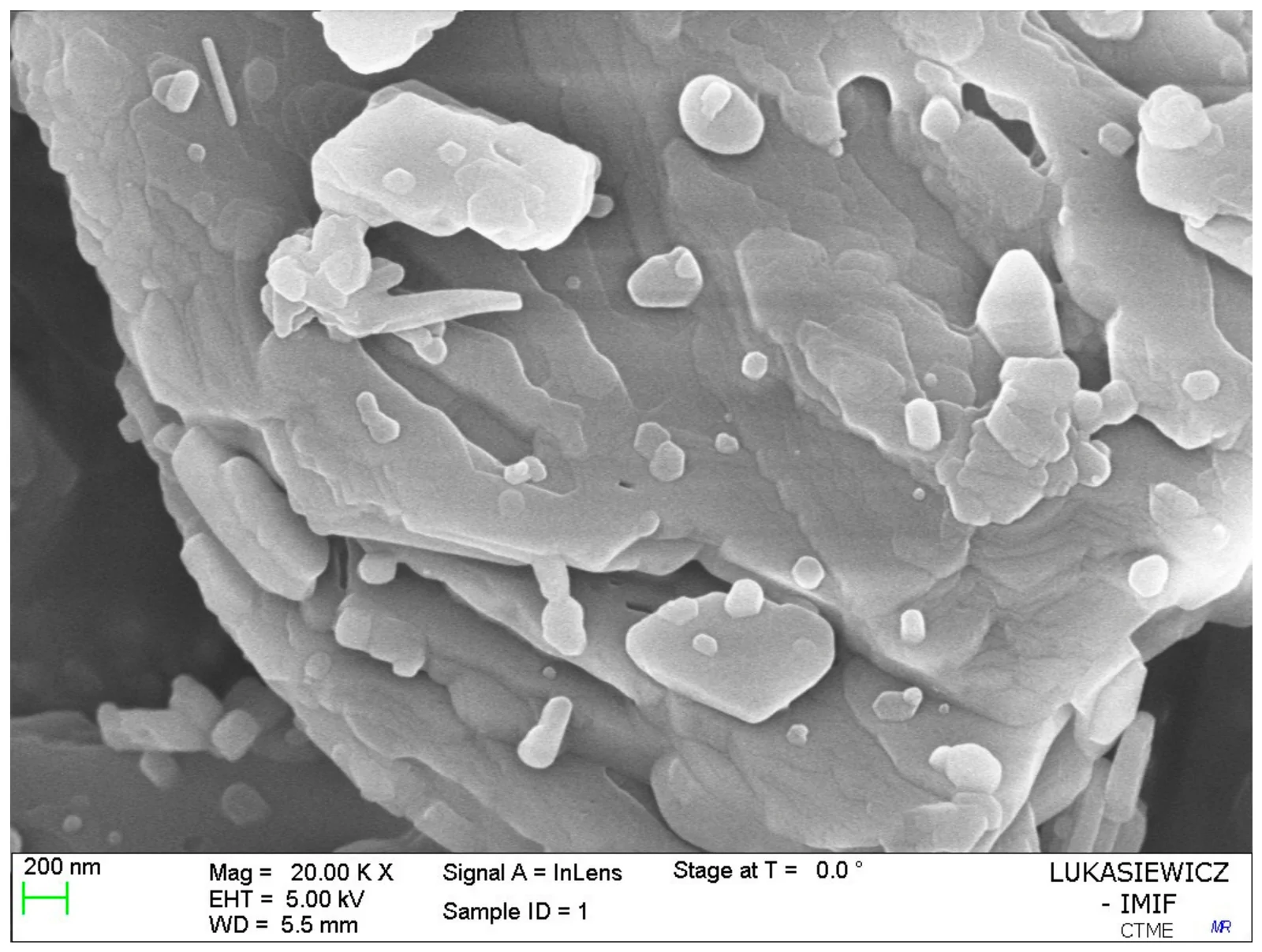
SEM image of the surface of unprocessed sodium naproxen—magnification: 20,000×.

**Figure 14:**
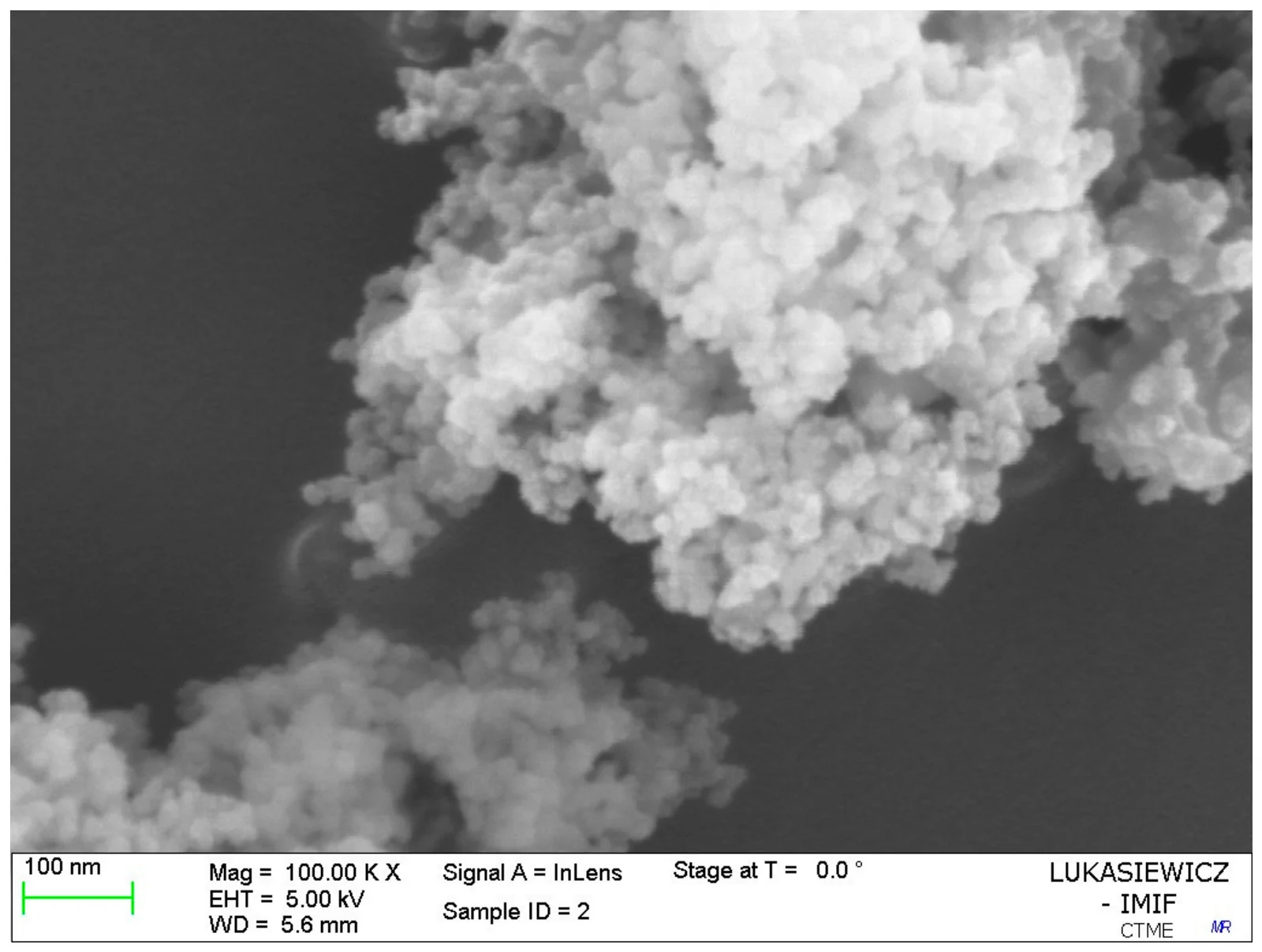
SEM image of Aerosil^®^ R972—magnification: 20,000×.

**Figure 15:**
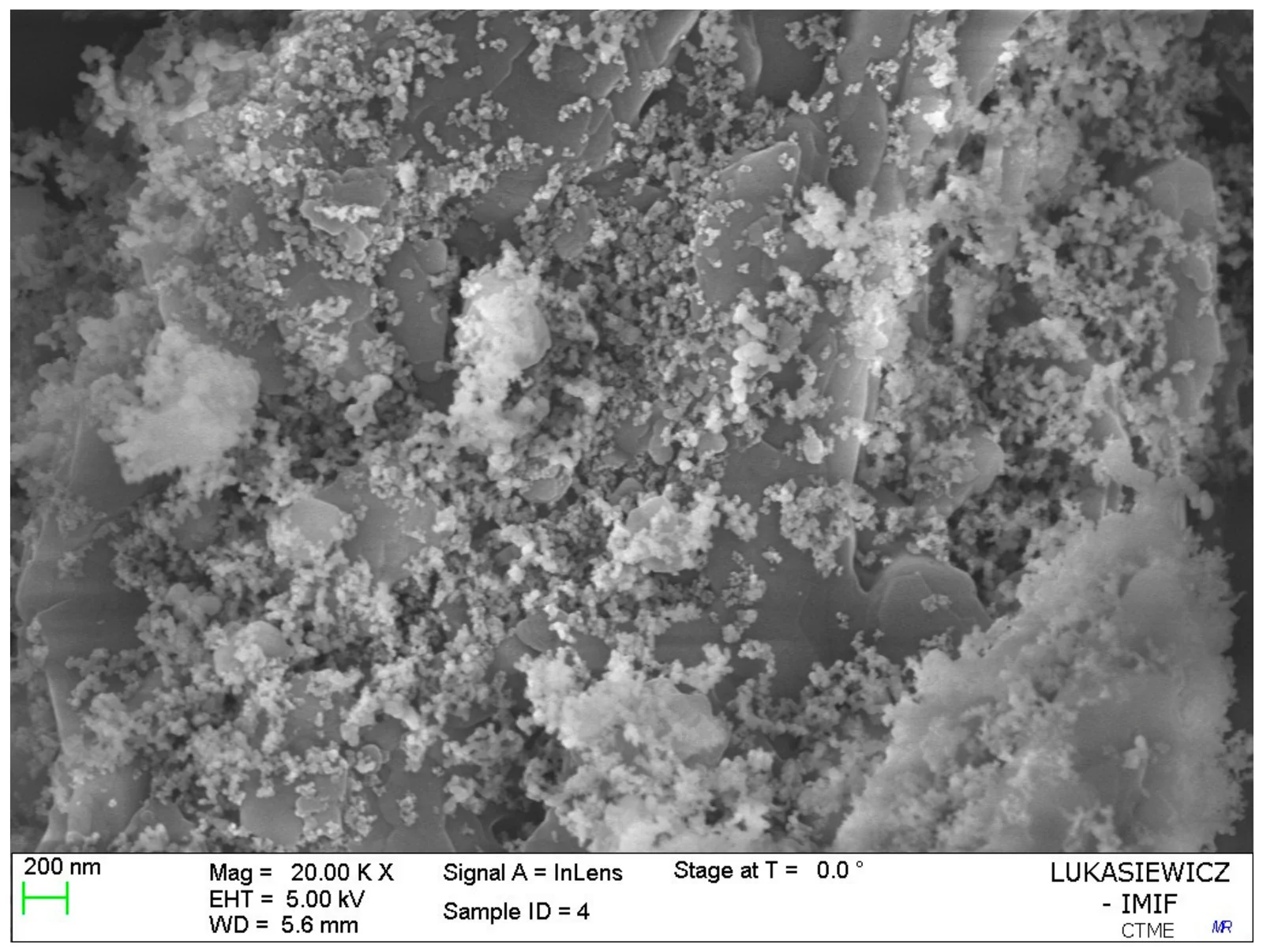
SEM image of the surface of sodium naproxen after interactive mixing (Sample 1)—magnification: 20,000×.

Based on the SEM images, it was found that the particles of unprocessed sodium naproxen are irregular and exhibit a smooth surface (Figure 13). After interactive mixing in the modified V-type tumbler mixer with the addition of 2% Aerosil^®^ R972 (Sample 1), partial coverage of the sodium naproxen particles with nanosilica (Figure 14) was observed (Figure 15).

**Figure 16:**
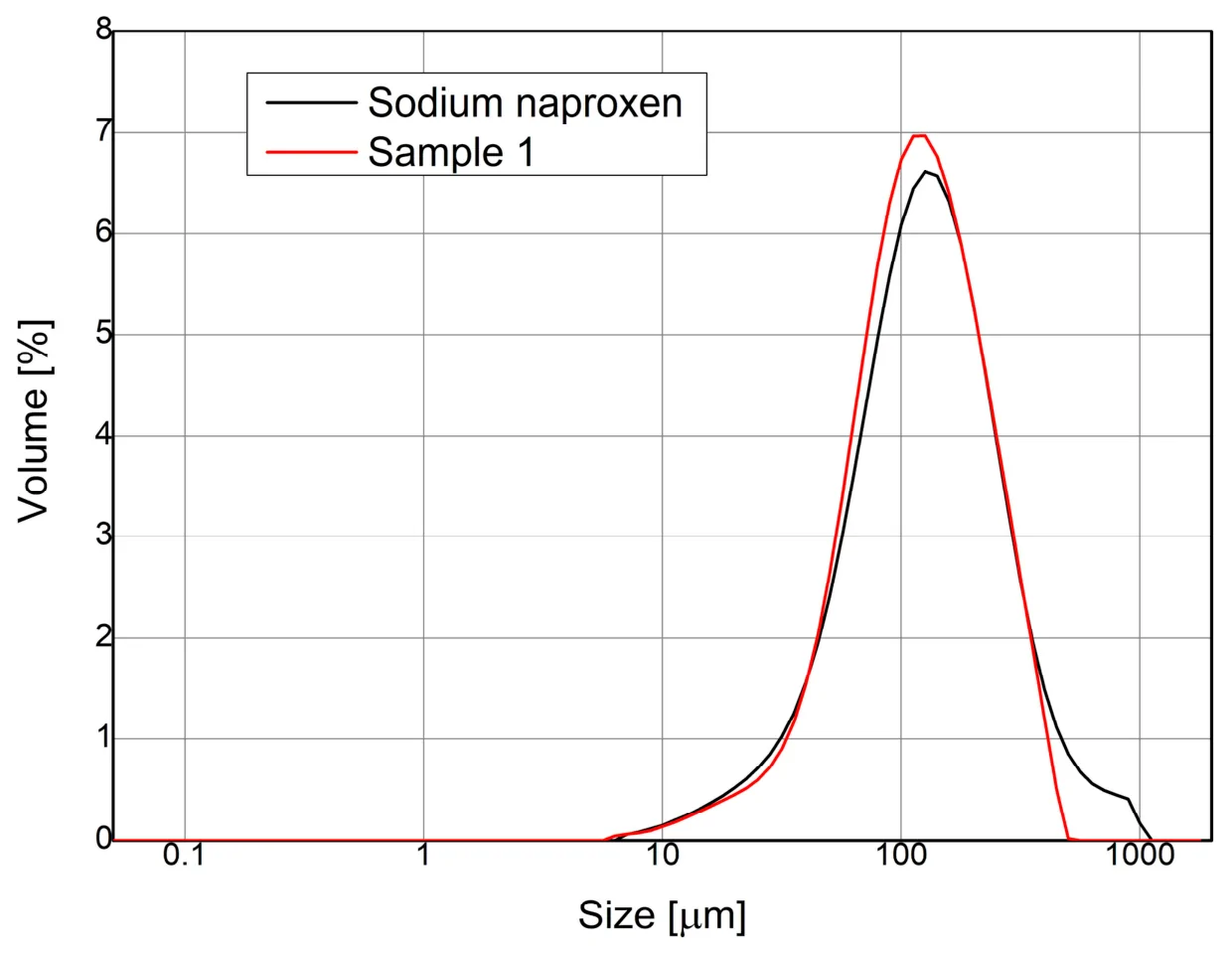
Particle size distribution of unmodified sodium naproxen and Sample 1 after interactive mixing.

The particle size distributions of unmodified and mechanically treated sodium naproxen were generally comparable (Figure 16). A reduction was mainly observed in the largest particle fraction (approximately 800–1000 µm), whereas the remaining particle size fractions were not substantially affected. This indicates that the treatment did not cause extensive particle fragmentation or a pronounced reduction in particle size, suggesting that the improvement in flowability was not primarily associated with particle size reduction.

## 4. Discussion

The observed improvements in powder flowability and mechanical behavior following the mechanochemical dry coating of sodium naproxen with 2% Aerosil^®^ R972 are consistent with established findings in powder engineering and pharmaceutical formulation. Dry coating with nanosilica generates a ball-bearing effect and introduces nanoscale surface roughness, reducing interparticle cohesion and improving powder flow [12,16,20]. Our study demonstrated a decrease in Carr indices and an increase in the flowability classification (*ffc* > 4) for Sample 1 at consolidation stresses above 65 kPa—results that closely mirror those obtained in studies on lactose monohydrate and ibuprofen [12,14,15].

Single-step coprocessing with glidants such as silica and magnesium stearate has been widely used to improve flow, content uniformity, and even drug release. In particular, dry-coated ibuprofen with nanosilica showed improved flow characteristics similar to our sodium naproxen formulation [20]. This confirms that silica-based coatings can be effective even for highly cohesive APIs.

The ball-to-powder ratio also affected the obtained flowability. In the preliminary experiments, ratios of 4:1 and 7:1 were compared, with the 7:1 ratio providing more favorable flowability results. Therefore, the 7:1 ratio was selected for the subsequent concentration study. The improved performance observed at the higher ratio may be related to a greater number of particle–media interactions and more effective mechanical energy transfer during mixing. However, these mechanisms were not directly measured in the present study and should therefore be considered as possible explanations for the observed effect.

The results show an improvement in the flowability of sodium naproxen with increasing Aerosil^®^ R972 concentration from 0.1 to 2%. This finding is consistent with previous studies showing that the addition of silica can increase particle surface coverage and reduce interparticle cohesive forces, thereby improving powder flowability [16,29]. Flowability continued to improve across the investigated concentration range, indicating that the effect had not yet reached a plateau under the studied conditions. However, the literature data suggest that further increases in silica concentration do not necessarily result in additional improvements in flowability, as excess silica may remain loosely attached to the particle surface and contribute to secondary agglomeration [25].

SEM analysis revealed changes in the surface morphology of the modified naproxen particles, consistent with partial surface coverage following the dry coating process. This finding aligns with prior studies where hydrophobic colloidal silica, such as Aerosil^®^ R972, preferentially adsorbed onto API surfaces under shear mixing, reducing van der Waals forces and electrostatic interactions [12,14].

Magnesium stearate showed a lower effect on the flowability of sodium naproxen compared with Aerosil^®^ R972 under the investigated conditions. This difference may be related to differences in particle size, surface properties, dispersion behavior, and the extent of deposition on the sodium naproxen particle surfaces. However, the present experiments do not allow the individual contributions of these factors to be quantitatively distinguished. Therefore, these factors should be considered as possible contributors to the observed difference rather than as established mechanisms.

One of the most notable findings was the reduced cohesion in Sample 1 at high consolidation stress (3.3–4.3 kPa), compared to 7.66 kPa for unmodified naproxen. This behavior aligns with reports on talc and starch, where dry coating with silica lowered interparticle adhesion, improving both flow and packing [17,30]. The comparison of the mechanical parameters indicates that the improvement in flowability was not associated with a substantial reduction in the internal friction angle. In fact, the modified material showed internal friction angles within a similar range to those of unmodified sodium naproxen over the investigated consolidation stresses. In contrast, a clear reduction in cohesion was observed for the modified material, particularly at the highest consolidation stress. These results suggest that the improved flowability is more likely associated with reduced interparticle cohesive forces than with a major change in the bulk internal friction behavior of the powder.

Importantly, this study confirms that high-performance dry coating can be achieved using a simple, low-speed V-type tumbler mixer, offering a potentially larger-scale and energy-efficient alternative to more complex systems like fluidized bed coaters or planetary mills [9,19]. This approach simplifies the implementation of dry coating in conventional pharmaceutical manufacturing lines.

These findings are particularly relevant to sodium naproxen processing, where the choice of mixing technology has previously been shown to affect powder homogeneity and tablet quality [31,32]. Earlier studies also demonstrated the use of more intensive mixing approaches, including vibratory milling, to modify the processing behavior of sodium naproxen formulations [31,32]. In contrast, the present results show that effective surface modification and improved flowability can be achieved using a relatively low mechanical energy input. This approach targets the interparticle interactions responsible for poor flowability while avoiding wet processing, drying, or intensive milling, highlighting its potential as a relatively simple and energy-efficient strategy for pharmaceutical powder processing.

Overall, the results indicate that the main benefit of mechanochemical dry coating is the modification of the consolidation-dependent cohesive behavior of sodium naproxen. The treatment did not substantially reduce the internal friction angle; instead, it reduced cohesion and limited the increase in powder-bed strength at higher consolidation stresses. The agreement between conventional flowability measurements, Jenike shear testing, and SEM observations supports the interpretation that Aerosil^®^ R972 modifies the surface interactions between sodium naproxen particles and thereby improves their flow behavior. Further studies should investigate the effect of the treatment on tabletability, dissolution, long-term storage, and scale-up, as well as the influence of coating concentration and mechanical energy on coating uniformity and performance.

The results demonstrate the potential of the proposed low-speed mechanically intensified system for improving powder flowability. Its potential for scale-up and application in pharmaceutical manufacturing requires further investigation. Further characterization of the processed powder would help distinguish the contribution of surface modification from potential attrition or fragmentation effects.

## 5. Conclusions

The results of this study demonstrate that mechanochemical dry coating of sodium naproxen with nanostructured silica (Aerosil^®^ R972) improves its flowability and processing performance. Among the investigated formulations, Sample 1, containing 2% silica and prepared using a modified V-type tumbler mixer, exhibited the most favorable properties. This formulation showed a substantial reduction in the compressibility index, angle of repose, and angle of difference, indicating improved powder mobility and packing behavior. Moreover, Jenike shear cell analysis revealed a pronounced reduction in cohesion at high consolidation stresses, while the internal friction angle remained within a comparable range to that of the unmodified material.

Surface characterization confirmed the deposition of silica particles on the sodium naproxen surface, demonstrating the effectiveness of the applied mechanochemical coating process. The obtained results are consistent with a reduction in interparticle cohesive interactions following dry coating, although the specific surface mechanism cannot be conclusively established from the present measurements.

An additional advantage of the proposed approach is the use of a relatively simple, accessible, and cost-effective mixing system, which offers compatibility with conventional pharmaceutical manufacturing processes. Furthermore, the presented mechanochemical surface modification method has been patented, highlighting its novelty and potential for industrial application in the processing and formulation of cohesive active pharmaceutical ingredients such as sodium naproxen.

## 6. Patents

The work presented in this manuscript is related to Polish Patent No. Pat.246299, entitled “Method for improving the flowability of sodium naproxen” (“Sposób poprawy zdolności płynięcia naproksenu sodu”), based on patent application P.440496 filed on 28 February 2022. The patent arose from research conducted in connection with the authors’ institutional activities at Rzeszów University of Technology.

The patent rights are distributed as follows: 10% is held by the Subcarpathian Innovation Center Ltd. (PCI), while the remaining 90% is equally divided among the three authors, who are named inventors and are affiliated with Rzeszów University of Technology.

## Figures and Tables

**Table 1:** Research plan.

Sample	Additive	Amount of Additive [% mass]	Ball-to-Powder Mass Ratio
Sodium naproxen	-	-	-
Sample 1	Areosil^®^ R972	2	7:1
Sample 2	Areosil^®^ R972	2	4:1
Sample 3	Areosil^®^ R972	1	7:1
Sample 4	Areosil^®^ R972	0.5	7:1
Sample 5	Areosil^®^ R972	0.1	7:1
Sample 6	Magnesium stearate	2	7:1

**Table 2:** Pre-shear consolidation and subsequent shear testing conditions used for the determination of the flow function *FF* and flowability index *ffc* of sodium naproxen using a Jenike shear cell.

Abbreviation	Pre-Shear Normal Load [kPa]	Normal Load During Shear Testing [kPa]
*σ_preA_*	20.2	*σ_nA_*_1_ 18.4 *σ_nA_*_2_ 17.5 *σ_nA_*_3_ 5.3
*σ_preB_*	22.2	*σ_nB_*_1_ 20.8 *σ_nB_*_2_ 18.4 *σ_nB_*_3_ 5.3
*σ_preC_*	27.6	*σ_nC_*_1_ 26.2 *σ_nC_*_2_ 24.4 *σ_nC_*_3_ 13.3
*σ_preD_*	33.3	*σ_nD_*_1_ 30.1 *σ_nD_*_2_ 27.6 *σ_nD_*_3_ 24.4

**Table 3:** Mechanical parameters of sodium naproxen.

	Pre-Shear Consolidation Load *σ_preA_* = 20.2 [kPa]	Pre-Shear Consolidation Load *σ_preB_* = 22.2 [kPa]	Pre-Shear Consolidation Load *σ_preC_* = 27.6 [kPa]	Pre-Shear Consolidation Load *σ_preD_* = 33.3 [kPa]
Angle of inclination of the stress boundary line [-]	0.812	0.768	0.781	0.607
Angle of internal friction *φ* [deg]	39.2	39.72	37.99	31.26
The point of intersection of the stress boundary lines with the tangential stress axis (cohesion) [kPa]	1.123	1.381	2.205	7.66
Maximum consolidation stress *σ*_1_ [kPa]	43.77	48.53	69.01	70.01
Uniaxial compressive strength *σ_c_* [kPa]	6.62	5.45	9.039	27.22
Flow index *ff_c_* [-]	6.61	8.90	7.635	2.572

**Table 4:** Mechanical parameters of Sample 1.

	Pre-Shear Consolidation Load *σ_preA_* = 20.2 [kPa]	Pre-Shear Consolidation Load *σ_preB_* = 22.2 [kPa]	Pre-Shear Consolidation Load *σ_preC_* = 27.6 [kPa]	Pre-Shear Consolidation Load *σ_preD_* = 33.3 [kPa]
Angle of inclination of the stress boundary line [-]	0.935	0.953	0.856	0.685
Angle of internal friction *φ* [deg]	43.06	43.62	40.57	34.42
The point of intersection of the stress boundary lines with the tangential stress axis (cohesion) [kPa]	1.438	1.476	2.051	4.292
Maximum consolidation stress *σ*_1_ [kPa]	59.596	63.226	70.727	71.419
Uniaxial compressive strength *σ_c_* [kPa]	6.624	6.891	8.911	16.289
Flow index *ff_c_* [-]	8.996	9.176	7.937	4.385

## Data Availability

The original contributions presented in this study are included in the article. Further inquiries can be directed to the corresponding author.
